# Visual assessment of [^18^F]flutemetamol PET images can detect early amyloid pathology and grade its extent

**DOI:** 10.1007/s00259-020-05174-2

**Published:** 2021-02-22

**Authors:** Lyduine E. Collij, Gemma Salvadó, Mahnaz Shekari, Isadora Lopes Alves, Juhan Reimand, Alle Meije Wink, Marissa Zwan, Aida Niñerola-Baizán, Andrés Perissinotti, Philip Scheltens, Milos D. Ikonomovic, Adrian P. L. Smith, Gill Farrar, José Luis Molinuevo, Frederik Barkhof, Christopher J. Buckley, Bart N. M. van Berckel, Juan Domingo Gispert, For the ALFA study, On behalf of the AMYPAD consortium

**Affiliations:** 1 Department of Radiology and Nuclear Medicine, Amsterdam UMC, Vrije Universiteit Amsterdam, De Boelelaan, 1117 Amsterdam, Netherlands; 2 Barcelonaβeta Brain Research Center (BBRC), Pasqual Maragall Foundation, Barcelona, Spain; 3 IMIM (Hospital del Mar Medical Research Institute), Barcelona, Spain; 4 Alzheimer Center and department of Neurology, Amsterdam UMC, Vrije Universiteit Amsterdam, De Boelelaan, 1117 Amsterdam, Netherlands; 5 Department of Health Technologies, Tallinn University of Technology, Tallinn, Estonia; 6 Radiology Centre, North Estonia Medical Centre, Tallinn, Estonia; 7 Nuclear Medicine Department, Hospital Clínic Barcelona & Biomedical Research Networking Center of Bioengineering, Biomaterials and Nanomedicine (CIBER-BBN), Barcelona, Spain; 8 Department of Neurology, University of Pittsburgh, Pittsburgh, PA USA; 9 Department of Psychiatry, University of Pittsburgh, Pittsburgh, PA USA; 10 Department of Geriatric Research Education and Clinical Center, VA Pittsburgh HS, Pittsburgh, PA USA; 11 GE Healthcare, Life Sciences, Amersham, UK; 12 Universitat Pompeu Fabra, Barcelona, Spain; 13 CIBER Fragilidad y Envejecimiento Saludable (CIBERFES), Madrid, Spain; 14 Centre for Medical Image Computing, and Queen Square Institute of Neurology, UCL, London, UK; 15 Department of Radiology and Nuclear Medicine, VU University Medical Center, De Boelelaan 1117, 1108 HV Amsterdam, The Netherlands; 16 Centro de Investigación Biomédica en Red de Bioingeniería, Biomateriales y Nanomedicina (CIBER-BBN), Madrid, Spain; 17 Alzheimer Prevention Program, BarcelonaBeta Brain Research Center (BBRC), C/ Wellington, 30, 08005 Barcelona, Spain

**Keywords:** Amyloid PET, [^18^F]flutemetamol, Regional visual read, Centiloid, Sensitivity, Neuropathology

## Abstract

**Purpose:**

To investigate the sensitivity of visual read (VR) to detect early amyloid pathology and the overall utility of regional VR.

**Methods:**

[^18^F]Flutemetamol PET images of 497 subjects (ALFA+ *N* = 352; ADC *N* = 145) were included. Scans were visually assessed according to product guidelines, recording the number of positive regions (0–5) and a final negative/positive classification. Scans were quantified using the standard and regional Centiloid (CL) method. The agreement between VR-based classification and published CL-based cut-offs for early (CL = 12) and established (CL = 30) pathology was determined. An optimal CL cut-off maximizing Youden’s index was derived. Global and regional CL quantification was compared to VR. Finally, 28 post-mortem cases from the [^18^F]flutemetamol phase III trial were included to assess the percentage agreement between VR and neuropathological classification of neuritic plaque density.

**Results:**

VR showed excellent agreement against CL = 12 (*κ* = .89, 95.2%) and CL = 30 (*κ* = .88, 95.4%) cut-offs. ROC analysis resulted in an optimal CL = 17 cut-off against VR (sensitivity = 97.9%, specificity = 97.8%). Each additional positive VR region corresponded to a clear increase in global CL. Regional VR was also associated with regional CL quantification. Compared to mCERAD_SOT_-based classification (i.e., any region mCERAD_SOT_ > 1.5), VR was in agreement in 89.3% of cases, with 13 true negatives, 12 true positives, and 3 false positives (FP). Regional sparse-to-moderate neuritic and substantial diffuse Aβ plaque was observed in all FP cases. Regional VR was also associated with regional plaque density.

**Conclusion:**

VR is an appropriate method for assessing early amyloid pathology and that grading the *extent* of visual amyloid positivity could present clinical value.

**Supplementary Information:**

The online version contains supplementary material available at 10.1007/s00259-020-05174-2.

## Introduction

Positron emission tomography (PET) imaging enables the in vivo assessment and quantification of amyloid-β (Aβ) neuritic plaque density, a pathological hallmark of Alzheimer’s disease (AD). In the clinical setting, the approved method for the assessment of amyloid pathology for supporting diagnosis using PET images is the visual read (VR), as described in the product labels of all currently registered amyloid PET tracers. To this end, VR has been validated against neuropathological determinations of amyloid burden [1–3]. However, it has been suggested that VR is a rather conservative method, as it was developed to indicate moderate-to-frequent plaques as evaluated using the CERAD classification [1, 4]. As a consequence, it is possible that this method misses the detection of early sparse amyloid accumulation, which could be of interest for detecting early amyloid abnormalities [5]. In addition, although several regions-of-interest (ROIs) are visually assessed as in accordance with the reader guidelines, generally only the final classification (i.e., negative/positive) is used in both research and clinical settings, omitting any information regarding the location and extent of amyloid pathology.

Differently than in the clinical routine, amyloid PET (semi-)quantification has mainly been used in the research setting to study both clinical and earlier (preclinical) populations. However, the considerable variability in choice of tracer and (semi-)quantitative methods across centers has challenged the comparability of quantitative outcomes. For that purpose, the recently proposed Centiloid scale has become an increasingly used approach for the harmonization of amyloid PET data. Local processing pipelines can be validated against the original Centiloid method, and tracer-specific metrics such as the standardized uptake value ratio (SUVr) can be converted to a common scale referred to as “Centiloid” (CL). The scale is anchored on [^11^C]PiB SUVr data and constructed such that CL = 0 represents the mean level of amyloid PET tracer uptake in young controls, while CL = 100 reflects the average signal observed in typical mild-to-moderate AD dementia patients [6]. This method has also been validated against neuropathological data by two independent studies [7, 8]. First, La Joie and colleagues (2019) demonstrated that the earliest detectable [^11^C]PiB signal occurred at CL = 12, and that a cut-off of CL = 24 best discriminated between subjects with none-to-low Aβ plaque burden and those with intermediate-to-high deposition [7]. Similar CL cut-off values were also identified by Amadoru and colleagues (2020), where CL = 10 was considered an optimal threshold for excluding neuritic plaques, while approximately CL = 21 successfully detected moderate-to-frequent plaque density [8]. In addition, a cut-off of CL = 12 was later also reported by Salvadó and colleagues (2019) to maximize the agreement between [^18^F]florbetapir and [^18^F]flutemetamol PET CL values from two different cohorts with respect to amyloid positivity as determined through CSF Aβ_42_ levels. Furthermore, when comparing to CSF p-tau/Aβ_42_ ratio levels as an indication of established AD pathology, the authors identified a cut-off of CL = 30 [9].

In contrast, studies using VR as the reference standard have reported significantly higher CL cut-offs (i.e., up to 42 CL) for determining amyloid abnormality [10–12]. This discrepancy is possibly due to substantial differences in the populations studied, with the number of preclinical individuals being limited or even absent in most VR studies. Preclinical AD participants are more likely to show low levels of amyloid burden in a focal manner [13] and therefore support more sensitive (lower) cut-offs than the specific (higher) ones identified from end-of-life subjects or typical clinical populations. Unfortunately, reports of regional VR are scarce and only available from clinical populations, where focal increase in signal has been visually observed in < 2% of individuals [14, 15]. However, as recent studies highlight the value of quantitative regional amyloid assessments in identifying focal amyloid pathology [16–18], performing systematic regional VR informed by the spatial-temporal evolution of amyloid pathology [19] could bring value to stage the progression of amyloid accumulation.

As stated in the strategic roadmap for an early diagnosis of AD framework, proper evaluation of VR performance in detecting early or focal amyloid deposition and establishing reader guidelines to facilitate such use remains incomplete [20]. Within this context, the aims of this study are twofold. First, we studied the agreement between VR- and CL-based classification of amyloid PET scans using previously proposed cut-offs for early and established amyloid accumulation. Secondly, we characterized and assessed the utility of regional VR positivity to stage amyloid burden across the AD *continuum*. To these ends, we pooled [^18^F]flutemetamol scans of two complementary cohorts that allowed us to cover both early and established pathology. The pooled cohort was intended to cover the full range of amyloid burden and to have a good representation of intermediate amyloid levels around proposed cut-offs for early amyloid accumulation. We also studied the inter- and intra-reader agreement in a subset of scans with mainly intermediate levels of amyloid burden, to assess the reproducibility of regional VR in the early stages of AD. Finally, we aimed to validate our results using an independent post-mortem data-set, in which (regional) VR was compared to neuropathological scores.

## Methods

### Subjects

Data from two cohorts were pooled in order to capture amyloid accumulation across the AD *continuum*; the ALFA+ cohort, which is a nested longitudinal long-term study of the ALFA (for ALzheimer’s and FAmilies) [21] and the Dutch Flutemetamol study from the Amsterdam Dementia Cohort (ADC) [22, 23]. The ALFA cohort was established as a research platform to characterize preclinical AD in 2743 cognitively unimpaired individuals, aged between 45 and 75 years old with increased risk for AD. The ALFA+ sub-study consists of participants enriched for family history of AD and APOE ε4 carriership and who underwent amyloid PET imaging. The first consecutive 352 participants of the ALFA+ study collected between March 2017 and January 2020 were included in this work. The ADC cohort consisted of cognitively impaired patients (mild cognitive impairment (MCI), AD dementia, and non-AD dementia (e.g., fronto-temporal dementia [FTD], dementia with lewy bodies [DLB]) who underwent standard dementia screening at the VU University Medical Center Amsterdam [22]. In total, 145 PET scans from ADC passed quality control for quantification (e.g., absence of significant lesions, brain parenchyma in field of view, and available high quality T1-weighted MRI) and were therefore included. Thus, a total of 497 [^18^F]flutemetamol scans were included in this study. Demographics are shown in Table 1.

**Table 1:** Demographics of the visual read cohorts

	Pooled (*N* = 497)	ALFA+ CU population (*N* = 352)	ADC Clinical Population (*N* = 145)	*p* value
Age (years)	61.7 ± 4.9	61.5 ± 4.6	62.2 ± 5.6	n.s.
Sex, Female (%)	281 (56.5%)	215 (61.1%)	66 (45.5%)	<0.01
MMSE	27.2 ± 3.5	29.2 ± 1.0	23.4 ± 3.4	<0.01
APOE ε4 carriership	280 (56.3%)	193 (54.8%)	87 (60.0%)	n.s.
Centiloid	18.7 ± 38.8	2.9 ± 17.2	56.8 ± 48.9	<0.01
VR+	141 (28.4%)	47 (13.4%)	94 (64.8%)	<0.01

*ALFA* ALzheimer’s and Families cohort, *ADC* Amsterdam Dementia Cohort, *CU* cognitively unimpaired, *MMSE* Mini-Mental Estate examination, *VR* visual read

The ALFA study and the PET sub-study (ALFA+) protocols have been approved by an independent Ethics Committee Parc de Salut Mar Barcelona and registered at Clinicaltrials.gov (ALFA Identifier: NCT02485730; PET sub-study Identifier: NCT02685969). Both studies have been conducted in accordance with the directives of the Spanish Law 14/ 2007, of 3rd of July, on Biomedical Research (Ley 14/ 2007 de Investigación Biomédica). The medical ethics review committee of the VU University Medical Center approved the Dutch Flutemetamol study (reference number: 2012/302).

### Amyloid PET acquisition, processing, and quantification

Scans from the ALFA+ (Siemens Biograph mCT scanner) and ADC (Gemini TF-64PET/CT scanner) cohort consisted of four frames (4 × 5 minutes) acquired 90–110 min post-injection of [^18^F]flutemetamol (ALFA+: 191 ± 14 MBq; ADC: 191 ± 10 MBq). All scans were pre-processed using a validated standard Centiloid pipeline and converted to the Centiloid scale [6]. To match the intrinsic resolutions between centers, we first smoothed the ALFA+ scans using an isotropic 3D Gaussian Filter with a 4-mm full width at half maximum (FWHM) to match the resolution of the PET scans from the joined cohort (see Sup. Figure 1 for example images before and after the resolution harmonization step). Subsequent steps were equal for both cohorts and have been previously reported [9]. Briefly, images were checked for motion and inter-frame registration was performed when necessary. Then, the four frames from the PET images were first averaged and co-registered to the corresponding T1-weighted scans. Then, the T1-weighted MRI scans were warped to standard space; the same warp was applied to warp the co-registered PET image. These procedures were performed in SPM12. Of note, different T1 protocols were used for each site. Acquisition details can be found in the supplementary material.

PET images were intensity normalized using the whole cerebellum as the reference region using the mask provided by the Centiloid method [6] (http://www.gaain.org/centiloid-project). Cortical Centiloid values were calculated using the standard target region and a previously calibrated conversion equation [9]. Based on their respective Centiloid values, scans were classified as amyloid negative (CL-: CL < 12), gray-zone (CL-GZ: CL = 12–30) or amyloid positive (CL+: CL > 30) [9]. In addition, regional standard uptake value ratios (SUVr) were extracted using the Desikan Killiany atlas [24] and converted to regional Centiloid units using the global conversion equation [6]. Five regions-of-interest (ROIs) were created to reflect the visual assessment guidelines: (1) frontal: rostral and caudal anterior cingulate cortex, medial and lateral orbitofrontal, superior frontal, frontal pole, rostral and caudal middle frontal, pars orbitalis, pars triangularis, and pars opercularis; (2) the precuneus (PC)/posterior cingulate cortex (PCC): precuneus, posterior cingulate cortex, and isthmus cingulate cortex; (3) lateral-parietal: superior parietal, supramarginal, and inferior parietal; (4) lateral temporal: transverse temporal, temporal pole and inferior, middle, and superior temporal cortex; and finally (5) striatum: putamen and caudate nucleus (Sup. Figure 2).

### Visual assessment of PET scans

All 497 [^18^F]flutemetamol scans were initially read by one reader (Reader 1, LEC), who was blinded to clinical details of the individuals, completed the training provided by GE Healthcare [25], and has experience in assessing >1000 scans. For the visual read, image maximum intensity was scaled to 90% of the pons signal using rainbow color scaling and transverse, sagittal, and coronal views were displayed using the software package Vinci 2.56 and assessed together with a T1-weighted MR scan to assist reading in the presence of atrophy in the visual assessment. Images were rated according to the read criteria as defined by the manufacturer, which included the visual assessment of 5 regions; frontal cortex, PC/PCC, lateral-parietal, lateral temporal, and striatum. In addition to regional reads, the final classification was also available, with images rated as either *positive* (VR+, unilateral binding in one or more cortical brain region or striatum) or *negative* (VR-, predominantly white matter uptake). Reader confidence of the final read was captured on a 5 point scale (1 very low confidence–5 very high confidence).

### Intra- and inter-reader agreement

Two additional readers (BvB and CB) were involved at a secondary step, where scans were independently selected (GS) to assess the intra- and inter-reader agreement, with an emphasis on the images with emerging levels of amyloid from the ALFA+ cohort. Scans were selected based on their initial VR assessment by Reader 1 and their Centiloid quantification. The selection criteria were (1) only one region assessed as amyloid positive based on VR (*N* = 19); (2) only the frontal and PC/PCC ROI were assessed as VR+ (*N* = 16); (3) VR assessment with low confidence (i.e., ≤ 3, *N =* 8); (4) discordant classification between VR and Centiloid (cut-off CL 12 [7, 9], *N =* 20); and (5) Centiloid values between 10 and 35 (*N =* 26). This resulted in the selection of 58 scans, as some fell into more than one inclusion category. In addition, 21 clearly negative and 21 clearly positive scans were also included to balance the sample, resulting in the final selection of 100 scans. Importantly, all readers (LEC, BvB, CB) were blinded to these selection parameters as well as to the initial (Reader 1) VR classification of the scans. BvB is a nuclear physician with considerable experience in reading [^18^F]flutemetamol scans and CB is a medical imaging expert employed at GE Healthcare.

### Post-mortem data-set

To further evaluate the utility of regional visual assessment of [^18^F]flutemetamol scans, we selected a sub-set of the post-mortem [^18^F]flutemetamol phase III study cases and compared the read of our three readers to the available neuropathological scores [26]. GS randomly selected a sample of 30 subjects from the original study, prioritizing for presence of MRI scans, shortest imaging-autopsy intervals, and intermediate levels of Aβ pathology as determined by CERAD. Also, different combinations of regional Aβ burden based on CERAD were represented. After selection, 2 cases were excluded due to severe vascular burden/lesions and severe atrophy, resulting in a final data-set for analyses of 28 cases. The readers were blinded to the selection. Demographics are shown in Table 2.

**Table 2:** Demographics of the post-mortem cohort

	All (*N* = 28)	Non-demented (*N* = 10)	Demented (*N* = 18)	*p* value
Age (years)	79.1 ± 9.3	75.2 ± 9.7	81.28 ± 8.5	.097
Sex, Female (%)	13 (46.4%)	3 (30%)	10 (55.6%)	.184
Delay PET imaging (days)	72.5 (111)	60.0 (311)	72.5 (104)	n.s.
VR+	15 (53.6%)	4 (40%)	11 (61.1%)	n.s.
Mean mCERAD_SOT_	1.08 (1.72)	0.09 (1.67)	1.15 (1.26)	.064

Age is shown in mean ± SD. PET delay and mCERAD_SOT_ are shown in median (IQR). VR: visual read. mCERAD_SOT_ modified CERAD standard of truth

We evaluated the VR results against a previously established neuropathological standard of truth (SOT) that was better suited for comparison with a PET study than the traditional CERAD-based classification. This modified CERAD standard of truth (mCERAD_SOT_) approach includes the assessment of neuritic plaque density in 8 neocortical regions (i.e., midfrontal lobe (MFL), middle and superior temporal gyrus (MTG/STG), inferior parietal lobe (IPL), anterior and posterior cingulate gyrus (ACG/PCG), precuneus (PRC), and primary visual cortex) and provides a continuous measure of pathology instead of a binary classification. Per region, a score of 0 = none (no plaques), 1 = sparse (1–5 plaques), 2 = moderate (6–19 plaques), or 3 = frequent (20+ plaques per 100× field of view [FoV]) was given. The scale midpoint of 1.5 represents the threshold between sparse and moderate categories. Thus, a mean score ≤ 1.5 was considered normal, while a mean score of >1.5 was considered abnormal for each region. If any one of the 8 regions was considered abnormal, i.e., any regional mCERAD_SOT_ was >1.5, the whole brain was considered abnormal or Aβ+. See Ikonovomic et al. (2016) for a detailed description of the methodology [27].

### Statistical analyses

Statistical Package for the Social Sciences (SPSS) version 26 was used for all statistical analyses, apart from the Kappa statistics, which were computed using R version 3.6.0. For the majority of cases (*N* = 397), only the assessment of Reader 1 was available for analysis. In cases where a majority VR was available (*N* = 100), this classification was used instead for both the global and regional analyses. Baseline demographics were described using simple descriptive statistical analyses.

#### Global visual read and global Centiloid

The aim of our first set of analyses was to compare global VR assessment to global Centiloid values. Kappa statistics were used to determine the agreement between Centiloid-based classification (cut-offs CL 12 and 30) and VR-based classification. In addition, the sensitivity, specificity, and Youden’s J index (sensitivity+specificity-1) of VR compared to CL were reported. Next, we aimed to derive the optimal Centiloid threshold using VR as standard of truth in an receiver operating characteristic (ROC) analyses, maximizing the Youden’s J Index.

#### Regional visual read and global and regional Centiloid

Our second group of main analyses aimed at comparing regional VR assessment and global and regional Centiloid values. First, differences in global CL quantification depending on the number of VR positive regions were assessed using Kruskal-Wallis test. Then, we assessed the difference in regional quantification (Centiloid and SUVr) by regional VR assessment using Wilcoxon test. Finally, the sensitivity and specificity associated with a maximized Youden index of regional VR compared to regional quantification were reported.

#### Patterns of regional visual read

Furthermore, as secondary analyses, we aimed to characterize VR stages based on the observed patterns of regional visual positivity. Chi-squared tests were used to assess the distribution of VR stages across CL groups and clinical diagnosis.

#### Intra- and inter-reader agreement

Finally, intra-reader agreement for Reader 1 and inter-reader agreement among the three readers regarding the final classification (i.e., negative/positive) was determined using Kappa statistics. Agreement was considered poor if *κ* was less than 0.20, satisfactory if *κ* was 0.21–0.40, moderate if *κ* was 0.41–0.60, good if *κ* was 0.61–0.80, and excellent if *κ* was more than 0.80. Reader agreement for regional visual read was assessed via percentage agreement, as the imbalance in negative/positive for certain regions affects the kappa statistic.

#### Visual read and neuropathological scores

First, we assessed the percentage agreement between global VR classification and neuropathological classification of neuritic plaque density (i.e., any region mCERAD_SOT_ > 1.5), reporting the number of true positives (TP), false positive (FP), false negatives (FN), and true negatives (TN). Then, we determined the percentage agreement between regional VR and regional mCERAD_SOT_ scores. Finally, we assessed the difference in continuous regional mCERAD_SOT_ score by regional VR assessment of negative/positive using a Wilcoxon test. More specifically, VR assessment of the frontal ROI was compared to neuropathological scores in the ACG and MFL, VR of PC/PCC ROI to PCG and PRC, VR of temporo-parietal to IPL, and VR of lateral temporal to MTG and STG.

## Results

### Relationship between Centiloid and global visual read

CL values ranged from −27.57 to 171.11, with a mean value of 19.82 (*SD* = 38.62) across the pooled dataset. After applying the previously established CL cut-offs of 12 and 30, 335 (64.4%) scans were classified as CL-, 44 (8.9%) as CL-GZ, and 118 (23.7%) as CL+. CL-GZ subjects were mostly cognitively unimpaired (*N* = 33, 75%), APOE ε4 carriers (*N* = 31, 70.5%), and distributed across a broad age range (*M* = 62.26,*SD* = 5.16, range = 49.6–70.6).

Across the pooled dataset, 141 (28.4%) scans were read as amyloid PET positive. Of the ALFA+ cohort (cognitively unimpaired population), 47 (13.4%) scans were read as amyloid positive, compared to 94 (64.8%) of the ADC cohort (cognitively impaired population). Visually amyloid PET positive scans had a significantly higher CL value than those visually negative (VR+: *M*_*CL*_ = 72.41, *SD*_*CL*_ = 35.09; VR-: *M*_*CL*_ = − 1.00, *SD*_*CL*_ = 8.06, *F* = 1378.18, *η*^2^ = 0.74, *p* < 0.01). In addition, within the VR+ group, quantitative amyloid burden was significantly different between the two cohorts (ALFA+: *M*_*CL*_ = 39.87, *SD*_*CL*_ = 17.77; ADC: *M*_*CL*_ = 88.67, *SD*_*CL*_ = 29.91, *F* = 106.15, *η*^2^ = 0.43, *p* < 0.01). In relation to CL groups, scans were read as positive in 0.3%/50.0%/100% of CL-/CL-GZ/CL+ cases, respectively.

### Visual read performance compared to Centiloid

First, we investigated the agreement between VR-based classification and Centiloid-based classification of amyloid positivity using the previously proposed CL cut-offs of 12 and 30. VR showed excellent agreement against both the lower bound (CL = 12, *κ* = .89, 95.2% [473/497]) and upper bound (CL = 30, *κ* = .88, 95.4% [474/497]) of the gray-zone cut-off, with a sensitivity/specificity of 85.9%/99.7% (PPV = 99.2%; NPV = 93.5%) and 100%/93.9% (PPV = 83.7%; NPV = 100%), respectively.

**Fig. 1:**
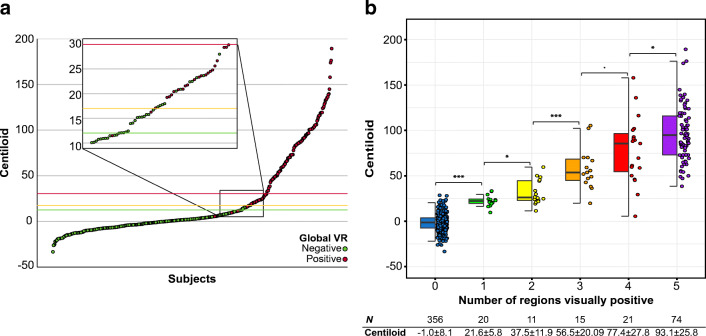
Visual read against global Centiloid. **a** Plots shows all 497 subjects ordered by global amyloid burden expressed in Centiloid units. The green line illustrates the CL = 12 cut-off as proposed by La Joie and colleagues (2019) based on post-mortem comparison and by Salvadó and colleagues (2019) based on CSF Aβ_42_. The red line illustrates the CL = 30 cut-off as previously proposed by Salvadó and colleagues compared to CSF p-tau/Aβ_42_, which was suggested to indicate the presence of established pathology. Finally, the orange line represents the optimal CL = 17 cut-off according the data-driven ROC analyses of this dataset using the Youden Index. **b** Centiloid values significantly increase per additional visually positive region. Post hoc analyses showed significant differences between all groups. *p* < 0.1; *p < 0.05; **p < 0.01; ***p < 0.001

Subsequently, we performed a ROC analysis with VR as the reference standard to assess whether the optimal CL cut-off in this independent dataset would fall within the previously reported 12–30 range. The overall agreement between VR and CL values was excellent (area under the curve [AUC] = 0.998; 95% CI: 0.996–1.0). A cut-off value of CL = 17, maximized the Youden’s Index (J = 0.956) and was associated with both very high sensitivity (97.9%) and specificity (97.8%) (Fig. 1a). See Sup. Table 1 for ROC results for all coordination points between 85% and 100% specificity. In addition, the sensitivity, specificity, and Youden Index as a function of CL can be found in Sup. Figure 3.

Finally, mean CL values showed a clear increase per additional positive VR region (*χ*^*2*^ = 303.71, df = 5, *p* < .001, Fig. 1b). Post hoc analyses revealed a statically significant difference in CL values between all consecutive groups based on number of regions read as positive and differences at trend level between 3 and 4 regions visually positive.

### Regional visual read and regional Centiloid

The PC/PCC and frontal regions were read positive most often (26.4% and 26.0%), followed by lateral temporal (20.3%), temporo-parietal (18.3%), and striatal region (17.9%). Isolated regional VR+ (one region only) occurred in only 20 subjects (4.0%), where the positive region was frontal in 9 subjects (1.8%) and PC/PCC in 11 subjects (2.2%). Out of 136 subjects that were PC/PCC VR+, 90.8% of them also were frontal VR+. Striatal VR+ always occurred with concomitant frontal VR+ (100%), while only .1% of striatal VR+ cases were not PC/PCC VR+, and around 15% of striatal VR+ cases were not temporo-parietal or lateral temporal VR+ (Sup. Table 2).

**Fig. 2:**
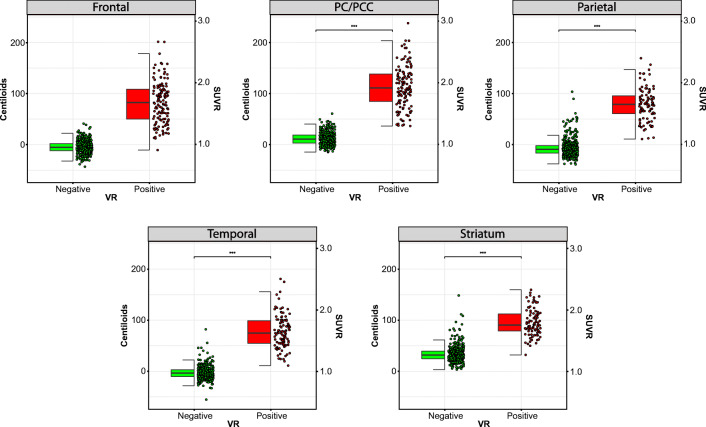
Regional visual read against regional quantification. Boxplots represent the regional visual assessment against regional amyloid burden, with quantification expressed in both Centiloid (y-axis left) and SUVR (y-axis right) units. PC/PCC: precuneus/posterior cingulate cortex; SUVR: standardized uptake value ratio; VR: visual read

Figure 2 shows for each VR ROI the regional amyloid burden quantified in both SUVr and CL units and stratified by VR status. For all regions, VR+ corresponded to significantly higher regional CL values (Frontal: *W* = 461; PC/PCC: *W* = 78; Parietal: *W* = 449; Temporal: *W* = 208; Striatum *W* = 791, *p* < 0.001, Sup. Table 3) and was accompanied with high sensitivity and specificity for all ROIs (frontal: sensitivity = 94.7%, specificity = 97.8%; PC/PCC: sensitivity = 100%, specificity = 96.2%; temporo-parietal: sensitivity = 96.8%, specificity = 95.5%; lateral temporal: sensitivity = 98.1%, specificity = 97.5%; striatum: sensitivity = 97.8%, specificity = 92.1%).

### Patterns of regional visual read

**Fig. 3:**
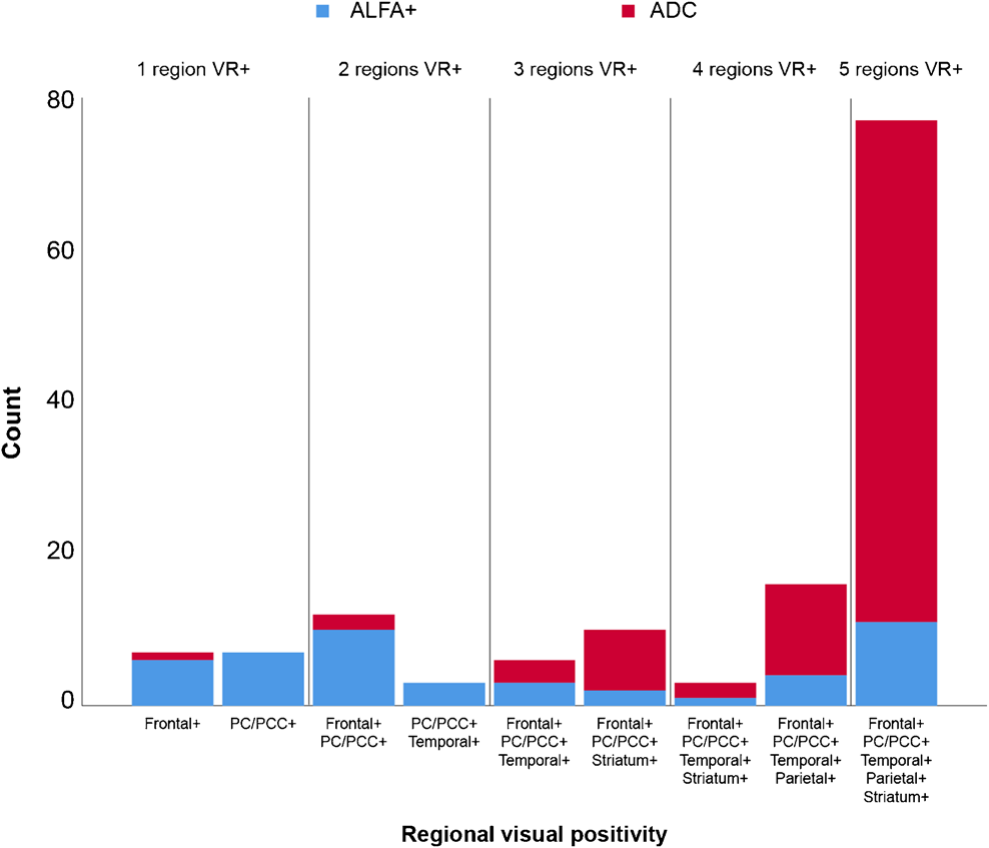
Patterns of visually positive regions. Bar graph represents number of subjects in each visual read group. In total, 10 combinations of regional amyloid positivity were observed. Blue represents the ALFA+ cognitively unimpaired subjects and red represents the ADC clinical cohort. PC/PCC: precuneus/posterior cingulate cortex; VR: visual read

Figure 3 shows the distribution of regional VR+, stratified per cohort. The distribution of subjects across the patterns suggests a general order of regions becoming visually amyloid positive; in case of one positive VR region, only the PC/PCC or frontal ROI was assessed as such (VR stage 1), most often (75%) followed by a combination of these regions being read as positive (VR stage 2). Then, further cortical and/or striatal visual positivity becomes apparent (VR stage 3). In the ALFA cohort, generally positivity beyond the PC/PCC and frontal ROIs was initially observed in the lateral temporal region, followed by the temporo-parietal regions, and finally the striatum. Early striatal involvement was more often reported in the cognitively impaired cohort. This could be the result of partial volume effects (i.e., atrophy) on the more lateral cortical regions, which is known to have a lesser effect on the striatal region.

**Fig. 4:**
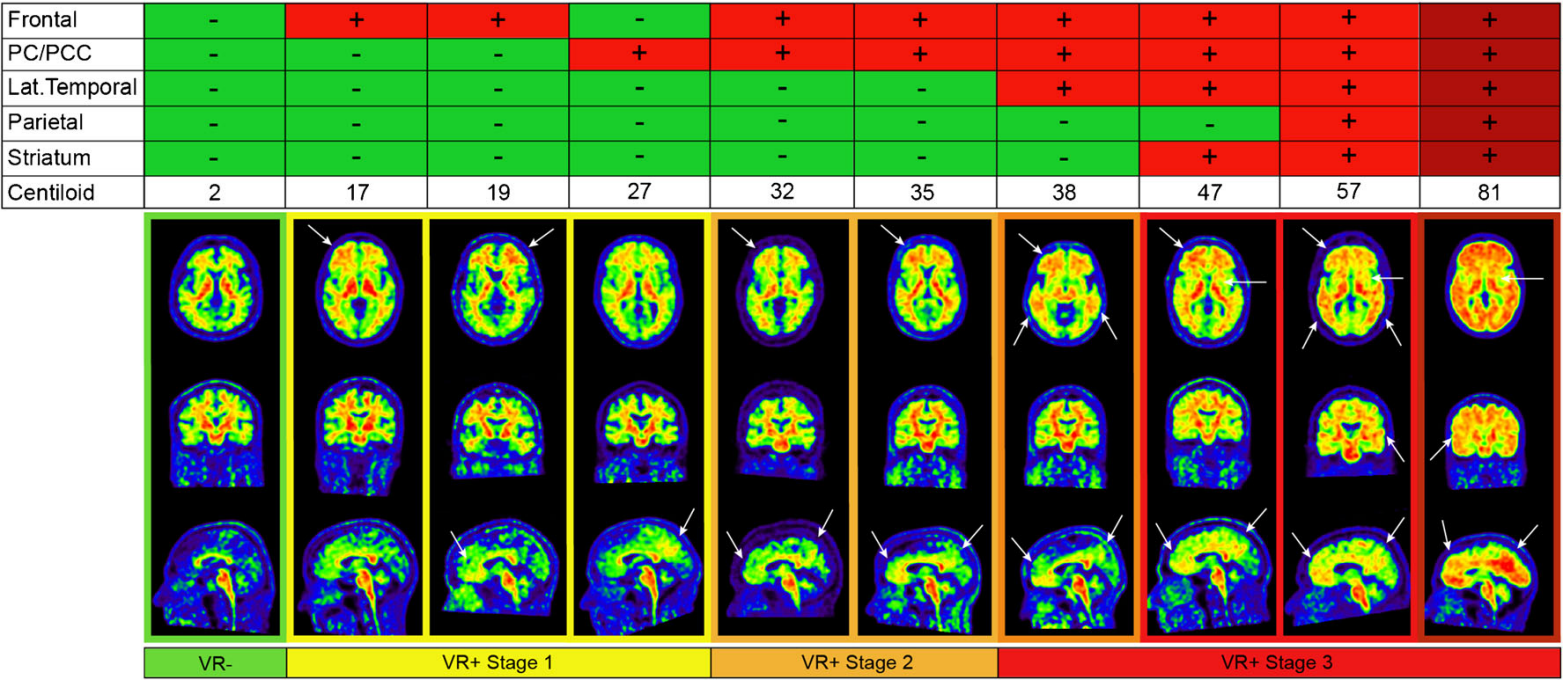
Example [^18^F]flutemetamol images. A series of 10 [^18^F]flutemetamol scans form the ALFA+ cohort ordered based on Centiloid values are shown. Upper panel illustrates which regions were visually assessed as positive. From top to bottom, axial, coronal, and sagittal images are provided. White arrows highlight specific regional amyloid uptake. Note, that the main differences between VR- (left panel) and early amyloid accumulation (second to fourth panel) can be observed basal frontally on the axial image and in the orbitofrontal and precuneal regions on the sagittal images. PC/PCC: precuneus/posterior cingulate cortex; VR: visual read

Mean CL values were significantly different between all VR stages (*H* = 302.55, *p* < .001), and the ROC analyses revealed the optimal CL cut-offs were CL = 16 (VR- vs. VR+ stage ≥1), CL = 22 (VR stage 0/1 vs. VR+ stage ≥2), and CL = 35 (VR stage 0/1/2 vs. VR+ stage 3), with good to excellent sensitivity/specificity (Table 3). Also, VR stages were associated with CL groups of low, gray-zone, and high amyloid burden (*χ*^2^ = 577.16, *p* < 0.01) and with clinical diagnosis (*χ*^2^ = 343.92, *p* < 0.01), which was made pre-disclosure of PET results. More details can be found in supplementary results and Sup. Figure 4. Figure 4 shows example images following this general pattern of visual amyloid positivity and their accompanying CL values.

**Table 3:** VR stages

	VR negative	VR+ stage 1	VR+ stage 2	VR+ stage 3
Number of subjects	356	20	9	110
Centiloid	− 1.0 ± 8.1	21.6 ± 5.8	35.4 ± 12.2	85.1 ± 28.3
CL cut-off*	n/a	16	23	35
Sensitivity*	n/a	97.8%	96.7%	97.3%
Specificity*	n/a	96.3%	97.8%	99.2%
Youden Index*	n/a	0.941	0.943	0.965
AUC*	n/a	.995 (.992-.999)	.992 (.992–1.00)	.996 (.992–1.00)

*Compared to lower stage(s)

### Intra- and inter-reader agreement

Based on the 100 pre-selected scans focused on the most difficult/borderline cases, intra-reader agreement of Reader 1 (LEC) was considered to be good (*κ* = .71). The overall agreement between the 3 readers was also good (*κ* = .75, 84%). The highest agreement was seen between Reader 1 and Reader 2 (*κ* = .78) and the lowest between Reader 2 and Reader 3 (κ = .72). Supplementary Table 5 shows all 100 cases ordered by CL burden and their final VR classification per reader. It shows that scans with a CL ~ 20 or higher burden are generally classified as VR+ across all readers. In addition, 4/9 of scans with a CL 17–20 were also classified as VR+ by at least 2 out of 3 readers. Importantly, reader agreement was high and comparable across all ROIs: frontal 74%, PC/PCC 84%, temporo-parietal 80%, lateral temporal 79%, and striatum 73%.

### Regional visual read and regional neuropathological scores

**Fig. 5:**
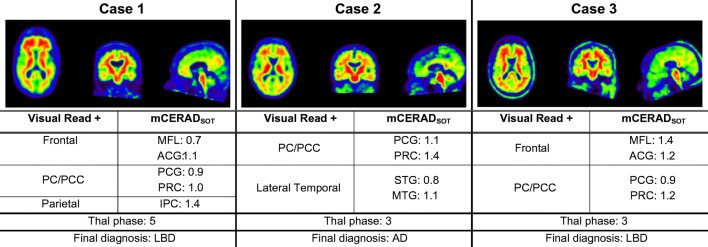
Visual read false positive cases. PC/PCC: precuneus/posterior cingulate cortex; MFL: midfrontal lobe; ACG: anterior cingulate gyrus; PCG: posterior cingulate gyrus; PRC: precuneus; IPC: inferior parietal cortex; STG: superior temporal gyrus; MTG: middle temporal gyrus; LBD: lewy body dementia; AD: Alzheimer’s dementia

Compared to mCERAD_SOT_-based classification (i.e., any region mCERAD_SOT_ > 1.5) of neuritic plaque density, VR classification was in agreement in 89.3% [25/28] of cases, with 13 TN, 12 TP, and 3 FP. Interestingly, all FP cases had a mean mCERAD_SOT_ above 1 and at least one region with a regional mean mCERAD_SOT_ of ≥1.3, indicating the presence of regional sparse-to-moderate neuritic amyloid plaques. In turn, only 1 TN cases had a similar pattern of neuropathological burden. In addition, these FP cases were reported to have a moderate to high burden of diffuse Aβ plaques, reflected in their Thal stage (i.e., 3–5). See Fig. 5 for a detailed description of these cases.

Compared to regional mCERAD_SOT_-based classification (i.e., regional mCERAD_SOT_ > 1.5), regional VR was in agreement in 75–89.3% of cases. Lower agreement was observed for the frontal and PC/PCC ROIs, as relatively more cases (11–14% vs. 0–7%) were classified as VR+ and did not have a mCERAD_SOT_ > 1.5, but rather a mCERAD_SOT_ between 1 and 1.5 (Sup. Table 6).

**Fig. 6:**
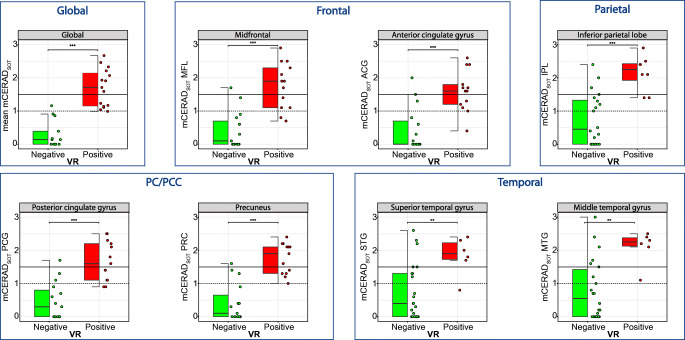
Visual read against neuropathological burden measured with mCERAD_SOT_. Boxplots represent the regional visual assessment (x-axis) against regional amyloid neuropathological burden (y-axis). Dotted line represents the cut-off for sparse-to-moderate (mCERAD_SOT_ = 1) and the full line the cut-off for moderate-to-frequent neuritic plaques (mCERAD_SOT_ > 1.5). MFL: midfrontal lobe; ACG: anterior cingulate gyrus; PCG: posterior cingulate gyrus; PRC: precuneus; IPC: inferior parietal cortex; STG: superior temporal gyrus; MTG: middle temporal gyrus; mCERAD_SOT_: modfied CERAD standard of truth; VR: visual read

Finally, both global and regional VR positivity were associated with significantly higher mean and regional neuropathological burden as measured with the mCERAD_SOT_ (Global: mean mCERAD_SOT_ *W* = 4; Frontal: MFL *W* = 13.5, ACG *W* = 21.5; PC/PCC: PCG *W* = 15.0, PRC *W* = 13.0; Parietal: IPL *W* = 12.0; Temporal: STG *W* = 19.5, MTG *W* = 16.0, all *p* < 0.001; Fig. 6, Sup. Table 6).

## Discussion

In the current study, we investigated the agreement between visual reads (VR) and Centiloid-based detection of early and established amyloid pathology and the utility of regional patterns of VR positivity for capturing the extent of amyloid burden beyond standard dichotomization. We found that VR-based classification performed by experienced readers is in high agreement with previously proposed quantitative Centiloid (CL) cut-offs of both early and established pathology. When using VR as the reference standard, we identified an optimal cut-off (CL = 17) well within the previously proposed gray-zone band of emerging amyloid pathology (CL = 12–30). In addition, there was a clear proportional relationship between the number of visually positive regions and increases in continuous CL burden, supporting the value of regional information in capturing the degree of amyloid burden. Furthermore, we observed that regional CLs were significantly higher in those regions assessed as positive by VR. The validity of this work is supported by our analyses in the post-mortem data-set, which showed a high agreement between VR-based and neuropathological-based classification of amyloid positivity, at both global and regional level. In fact, these results suggest that VR could capture the presence of sparse-to-moderate neuritic plaques and substantial diffuse Aβ plaques.

In recent years, great emphasis has been put on improving the early identification of amyloid pathology. In a clinical trial setting, amyloid PET is increasingly used as subject selection tool and criteria are often based on visual assessment in accordance with the product label [28]. As drug interventions move towards secondary prevention and preclinical populations [17, 29], ensuring the early detection of brain amyloid by means of VR could become crucial. In turn, identification of early pathology might also be of value for clinical use, considering the current interest in preclinical AD pathology in the memory clinic setting [22, 30]. Previous work in the clinical setting using VR as the reference to determine CL cut-offs have reported a broad range of relatively high thresholds (24–42 CL) [7, 8, 10, 11, 31], while a quantitative burden of CL > 21 has already been found to correspond to established pathology [7, 8] based on post-mortem samples. While this contrast may suggest a suboptimal VR sensitivity, the limited number of cases with emerging amyloid pathology in those studies may have limited the assessment of the true sensitivity of VR. In this work, >70% of the clinical dataset were cognitively unimpaired subjects, who are more likely to show subtle amyloid pathology, and indeed 44 subjects showed tracer uptake values within the gray-zone of amyloid burden. Therefore, this study was uniquely enriched with subjects around the expected threshold band, resulting in an observed VR-based CL cut-off of 17, with excellent sensitivity and specificity. Importantly, previous work from Su and colleagues (2018) showed that the CL quantification and consequently cut-offs vary based on local processing pipelines. They demonstrated that for a given criterion (i.e., 95% specificity), the resulting CL cut-offs ranged between 6 and 12, illustrating the value of a confidence interval in cut-off determinations [32]. Therefore, even though the optimal cut-off is calculated to be 17 in the current work, a range between 14 and 20 CL could be expected depending on the particular pipeline implementation. Nonetheless, our ROC analysis showed that this range of CL cut-offs is accompanied by a high Youden’s Index (i.e., >0.9) (Sup. Table 1 and Sup. Figure 3), suggesting that such deviations in cut-offs may not significantly affect classification performance.

In addition to enriching the dataset, another unique characteristic of the study is the experience of the readers, who were familiar with research scans showing early amyloid deposition. Therefore, these readers may have been more confident than others when reading a scan with focal deposition as amyloid-positive, also contributing to a lower CL cut-off than previously reported from routine clinical cohorts. Indeed, both the intra- and inter-reader agreement were relatively high compared to previous work in a similar population [13, 33], further illustrating the experience of the readers. The inter-reader agreement analyses also showed that most of the scans with a quantitative burden above 17 CL were assessed as positive by at least 2 out of 3 readers. In addition, all readers consistently assigned visual positivity to scans with a quantitative burden of >20 CL, which is similar to previous work using one experienced reader [8]. Importantly, the regional read agreement was highly similar to the global classification agreement, supporting its utility for routine use.

The most commonly reported visually positive regions in this study, either isolated or in combination with other regions, were the precuneus and the (medial orbito) frontal cortex, including the anterior cingulate. As illustrated in Fig. 4, the sagittal plane seems to be optimal for visually detecting emerging amyloid pathology, as both these regions can be easily assessed using this orientation. While the VR [^18^F]flutemetamol guidelines for the PC/PCC ROI already state the sagittal plane as the primary orientation for assessment, it is considered as *supportive* for the frontal ROI, where the primary orientation is the axial view. Although the axial view is an appropriate orientation to assess basal frontal uptake (example case Fig. 4 2nd panel), the sagittal view allows for the specific assessment of the medial orbitofrontal cortex (example case Fig. 4 3rd panel). The importance of these two regions is further supported by several articles in the field of amyloid staging, where PET-based regional quantitative burden has been used to identify a general order of regional involvement [16, 34]. Also, a recent review points to the importance of medial cortical regions in optimizing amyloid PET sensitivity [19]. It is important to realize that the sensitivity of medial regions is partly influenced by signal properties of PET imaging: due to their proximity to white matter and the additional gray matter signal spill-in from the contralateral hemisphere, medial regions are more frequently classified as positive in PET imaging compared to lateral counterparts, while levels of pathology are comparable [15]. This could explain why the overall quantitative burden as measured in CL units could already be relatively high, while visually the scan displays only focal deposition (example case Fig. 4, 4th panel). Therefore, this isolated or early amyloid deposition which is most often visually observed in medial cortical regions could already reflect more extensive but undetected pathological burden throughout the brain. Indeed, our post-mortem results seem to support this hypothesis, as while VR positivity in, e.g., the PC/PCC ROI corresponds to neuropathological scores indicative of sparse-to-moderate neuritic plaques, VR positivity in the lateral regions is associated with higher pathological burden. Considering that readers are now confronted with research scans more often, this knowledge can be useful to guide their assessment of early accumulation.

Beyond traditional dichotomized classification of amyloid negative/positive, reporting the number of VR+ regions to stage the severity of amyloid burden could be of value. We showed that the extent of amyloid burden in terms of number of visually positive regions and the derived VR stages related in a proportional manner to increasing CL values. More specifically, while 1 or 2 (VR+ Stages 1 and 2) visually positive regions are in line with previously proposed CL threshold of either emerging (~12 CL) or more established (~30 CL) amyloid pathology [7–9], 3 or more visually positive regions (VR+ Stage 3) are in line with CL values suggested to reflect clinical meaningful amyloid pathology (Table 2). For example, in addition to a cut-off of 26 CL for predicting clinical progression, Hanseeuw and colleagues [12] showed that in non-demented memory clinic patients, a cut-off of 42 CL was optimal in predicting progression to dementia over a period of 6 years. This last cut-off corresponds well to the observed CL burden associated with 3 visually positive regions in this work. In addition, Amadoru and colleagues [8] concluded that a CL burden of >50 best confirmed a clinicopathological diagnosis of AD and the mean quantitative burden of patients with AD dementia can vary from 84 CL [35] to 100 CL [6]. These values are in agreement with what we observed in scans with 4 or 5 visually positive regions. Together, these correspondences indicate the extent of amyloid burden can be visually assessed and future work should determine whether it conveys prognostic information. Longitudinal data collection is necessary to determine whether regional VR has similar prognostic value as compared to quantification. Currently, the 4-year follow-up including both amyloid PET acquisition and cognitive measures of the ALFA+ cohort is being collected in collaboration with the AMYPAD Consortium [17], which will enable analyses to assess the value of regional VR in a longitudinal setting.

This work shows that VR is both sensitive enough to capture early pathology for clinical trials aimed at secondary prevention, and useful for staging a subject according to their regional amyloid burden. However, several aspects are important in order to perform the regional visual assessment in an accurate manner. The following observations can be considered when performing visual assessment of [^18^F]flutemetamol PET images:

1. Especially in the research context, readers could benefit from focusing on the medial regions, using the sagittal view as the primary orientation for visual assessment of early amyloid pathology. Of note, a proper alignment of the images is key to ensure accurate assessment of the gray rather than the white matter signal. A suitable pivot point for all rotations is the inferior tip of the posterior corpus callosum at the junction of the hemispheres.
2. In future clinical routine, documenting the *extent* of amyloid burden could be a valuable asset in addition to the final read classification of amyloid negative/positive.

Of note, the generalizability of these results remains to be investigated in light of differences between tracers with respect to reading “signs,” use of different color scales [36], and possibly distinct influence of WM uptake in the distortion of the PET signal in medial regions [15].

Some limitations of this work should be considered. First, the mean age of our clinical cohort (ADC) is relatively low. This is due to the fact that the Alzheimer Center Amsterdam is a specialized tertiary referral center, which assesses a more atypical and generally younger patients [22]. Second, it should be noted that the clinical diagnosis in this cohort was made pre-PET disclosure; thus, any discrepancies between diagnosis and the presence of amyloid pahtology could also reflect misdiagnosis. Also, the extent of amyloid burden should be considered in combination with clinical disease severity, as the presence of early amyloid pathology in patients with dementia might reflect co-pathology rather than dementia due to AD. This will be investigated within in AMYPAD consortium, where regional VR is captured for all patients participating in the Diagnostic and Patient Management Study (DPMS) [30]. Third, T1 sequences for this cohort originate from multiple scanners as part of the clinical routine, which could have introduced noise to the quantitation. However, recent work has shown that the amount of variance introduced by this methodological aspect is within the physiological scan-rescan range and lower than the within-ROI variability, suggesting a minor impact on amyloid PET studies [37]. Fourth, the majority of visual assessments in the clinical cohort was performed by one reader, while a majority visual read was available for those cases displaying emerging or focal amyloid deposition. Nonetheless, since scans with a single read represented more the extremes of the quantitative spectrum, it is likely that these cases were mostly clearly negative or positive and therefore we could assume that an additional read would not have significantly affected the results. Finally, the post-mortem data-set used in this work was only a subset of the previously reported [^18^F]flutemetamol Phase III trial. However, by prioritizing the inclusion of cases with non-extreme CERAD neuritic plaque scores, we believe to have demonstrated with sufficient information the validity of a regional visual read and its ability to capture early pathology.

## Conclusion

Visual assessment of amyloid PET scan is capable of detecting early amyloid pathology and regional visual positivity captures its extent. More specifically, we have shown that the threshold for visual read is 17 CL, with a sensitivity and specificity of ~98% and corresponds to neuropathological scores indicative of sparse-to-moderate neuritic plaques in specific brain regions. These two aspects could be highly valuable in both a research/clinical trial and future clinical routine setting. Further work should investigate the prognostic value of regional VR compared to quantitation and the comparability between amyloid radiotracers.

## Supplementary Information

ESM 1 (DOCX 916 kb)
