# Supplementary material for: Visual assessment of [18F]flutemetamol PET images can detect early amyloid pathology and grade its extent
Source: Eur J Nucl Med Mol Imaging. 2021 Feb 22;48(7):2169–82. doi: 10.1007/s00259-020-05174-2 (PMC8175297; doi:10.1007/s00259-020-05174-2)
Supplement: Supplementary file 1 — (DOCX 916 kb) [file 259_2020_5174_MOESM1_ESM.docx]

**Supplementary Material**

**T1 sequence parameters**

For ALFA+ cohort, the T1-weighted 3D-TFE sequence was acquired in a Philips 3 T Ingenia CX scanner with a voxel size of 0.75 × 0.75 × 0.75mm3, FOV 240 × 240 × 180 mm3, sagittal acquisition, flip angle 8°, TR = 9 .9ms, TE = 4 .6ms, TI = 900 ms.

For the ADC cohort: T1 settings differed as the sequence was obtained from several scanners during clinical routine. Nonetheless, all images were visually checked to ensure sufficient quality for PET processing efforts.

ADC imaging data were acquired with six different scanners, including three 1.5T and three 3T. The three MRI 3T scanners used an 8-channel head coil and were: 1) single GE Signa 3T using a three dimensional (3D) T1-weighted sagittal fast spoiled gradient echo (FSPGR) sequence TR = 8, TE = 3, TI = 459, flip angle (FA) = 12°, 0.98 x 0.98 x 1.00 mm voxels); 2) Toshiba Titan 3T scanner with 3D sagittal fast field echo (FFE) sequence (TR = 9, TE = 3, TI = 800, FA = 7°, 1.00 x 1.00 x 1.00 mm voxels) and 3) Philips Ingenuity Time-of-Flight PET/MRI-scanner with a 3D sagittal turbo field echo (TFE) sequence (TR = 7.9 ms, TE = 4.5 ms, FA° = 8, 1.00 mm x 1.00 mm x 1.00 mm voxels) [1]. The three MRI 1.5T scanners were: 1) Siemens impact 1.5T in coronal plane (TR = 15 ms, TE = 7 ms, (TI) 300 ms, FA = 15°, voxel size 1×1×1.5 mm); 2) Siemens AVANTO 1.5T in coronal plane (TR = 2700 ms, TE = 5.2 ms, TI = 950 ms, FA = 8°, voxel size 1×1×1.5 mm) and; 3) GE SIGNA 1.5T in sagittal plane, (TR = 12.4 ms, TE =5.17 ms, TI = 450 ms, FA = 12°, voxel size 0.98×0.98×1.5 mm) [2].

**Sup. Table 1. ROC coordination points**

| **Centiloid cut-off** | **Sensitivity** | **Specificity** | **Youden Index** |
| --- | --- | --- | --- |
| 7,0223 | 1,000 | 0,851 | 0,851 |
| 7,0556 | 1,000 | 0,854 | 0,854 |
| 7,0661 | 1,000 | 0,857 | 0,857 |
| 7,1060 | 1,000 | 0,860 | 0,860 |
| 7,1690 | 1,000 | 0,862 | 0,862 |
| 7,2216 | 1,000 | 0,865 | 0,865 |
| 7,4793 | 1,000 | 0,868 | 0,868 |
| 7,7181 | 1,000 | 0,871 | 0,871 |
| 7,7686 | 1,000 | 0,874 | 0,874 |
| 7,8905 | 1,000 | 0,876 | 0,876 |
| 7,9931 | 1,000 | 0,879 | 0,879 |
| 8,0658 | 1,000 | 0,882 | 0,882 |
| 8,1295 | 1,000 | 0,885 | 0,885 |
| 8,1891 | 1,000 | 0,888 | 0,888 |
| 8,3060 | 1,000 | 0,890 | 0,890 |
| 8,4089 | 1,000 | 0,893 | 0,893 |
| 8,4618 | 1,000 | 0,896 | 0,896 |
| 8,5406 | 1,000 | 0,899 | 0,899 |
| 8,6547 | 1,000 | 0,902 | 0,902 |
| 8,9697 | 1,000 | 0,904 | 0,904 |
| 9,4793 | 1,000 | 0,907 | 0,907 |
| 9,7720 | 1,000 | 0,910 | 0,910 |
| 9,8635 | 1,000 | 0,913 | 0,913 |
| 9,9972 | 1,000 | 0,916 | 0,916 |
| 10,1985 | 1,000 | 0,919 | 0,919 |
| 10,5045 | 1,000 | 0,921 | 0,921 |
| 10,8076 | 1,000 | 0,924 | 0,924 |
| 10,9267 | 1,000 | 0,927 | 0,927 |
| 11,0445 | 1,000 | 0,930 | 0,930 |
| 11,2509 | 0,993 | 0,930 | 0,923 |
| 11,4601 | 0,993 | 0,933 | 0,925 |
| 11,6435 | 0,993 | 0,935 | 0,928 |
| 11,8605 | 0,993 | 0,938 | 0,931 |
| 12,0527 | 0,993 | 0,941 | 0,934 |
| 12,0961 | 0,993 | 0,944 | 0,937 |
| 12,1097 | 0,993 | 0,947 | 0,940 |
| 12,2437 | 0,993 | 0,949 | 0,942 |
| 12,5805 | 0,993 | 0,952 | 0,945 |
| 13,0906 | 0,993 | 0,955 | 0,948 |
| 13,4457 | 0,986 | 0,955 | 0,941 |
| 13,5376 | 0,986 | 0,958 | 0,944 |
| 13,7519 | 0,986 | 0,961 | 0,946 |
| 13,9486 | 0,979 | 0,961 | 0,939 |
| 14,1715 | 0,979 | 0,963 | 0,942 |
| 14,9743 | 0,979 | 0,966 | 0,945 |
| 15,9022 | 0,979 | 0,969 | 0,948 |
| 16,4539 | 0,979 | 0,972 | 0,951 |
| 16,8196 | 0,979 | 0,975 | 0,953 |
| 16,9684 | 0,979 | 0,978 | 0,956 |
| 17,0093 | 0,972 | 0,978 | 0,949 |
| 17,1314 | 0,972 | 0,980 | 0,952 |
| 17,2831 | 0,965 | 0,980 | 0,945 |
| 17,9903 | 0,965 | 0,983 | 0,948 |
| 18,8141 | 0,957 | 0,983 | 0,941 |
| 19,0012 | 0,950 | 0,983 | 0,934 |
| 19,1261 | 0,950 | 0,986 | 0,936 |
| 19,6113 | 0,950 | 0,989 | 0,939 |
| 20,0944 | 0,950 | 0,992 | 0,942 |
| 20,2979 | 0,950 | 0,994 | 0,945 |
| 20,3879 | 0,943 | 0,994 | 0,938 |
| 20,5778 | 0,936 | 0,994 | 0,931 |
| 20,9670 | 0,929 | 0,994 | 0,923 |
| 21,6131 | 0,922 | 0,994 | 0,916 |
| 22,5359 | 0,915 | 0,994 | 0,909 |
| 23,1547 | 0,908 | 0,994 | 0,902 |
| 23,5574 | 0,901 | 0,994 | 0,895 |
| 24,4874 | 0,894 | 0,994 | 0,888 |
| 25,2015 | 0,887 | 0,994 | 0,881 |
| 25,2974 | 0,879 | 0,994 | 0,874 |
| 25,5570 | 0,879 | 0,997 | 0,877 |
| 25,9478 | 0,872 | 0,997 | 0,870 |
| 26,7816 | 0,865 | 0,997 | 0,862 |
| 27,5323 | 0,858 | 0,997 | 0,855 |
| 27,7540 | 0,851 | 0,997 | 0,848 |
| 28,5343 | 0,844 | 0,997 | 0,841 |
| 29,1934 | 0,844 | 1,000 | 0,844 |
| 7,0223 | 1,000 | 0,851 | 0,851 |
| 7,0556 | 1,000 | 0,854 | 0,854 |
| 7,0661 | 1,000 | 0,857 | 0,857 |

**Sup. Table 2. Co-occurrence regional visual positivity**

| **VR+** | **PC/PCC** | **Frontal** | **Temporal** | **Parietal** | **Striatum** |
| --- | --- | --- | --- | --- | --- |
| **PC/PCC (*N*= 131)** | x | 119 (90.8%) | 101 (77.1%) | 91 (69.5%) | 888 (67.2%) |
| **Frontal (*N*=129)** | 119 (92.2%) | x | 100 (77.5%) | 91 (70.5%) | 89 (69.0%) |
| **Temporal (*N*= 101)** | 101 (100%) | 100 (99.0%) | x | 91 (90.1%) | 78 (77.2%) |
| **Parietal (*N*= 91)** | 91 (100%) | 91 (100%) | 91 (100%) | x | 74 (81.3%) |
| **Striatum (*N*= 83)** | 88 (98.9%) | 89 (100%) | 78 (87.6%) | 74 (83.1%) | x |

**Sup. Table 3. Regional Centiloid burden**

| **ROI** | **VR-** | **VR+** | ***p*** |
| --- | --- | --- | --- |
| **Frontal** | -5.3 (13.9) | 82.3 (58.6) | <0.001 |
| **PC/PCC** | 10.6 (15.7) | 111.0 (53.7) | <0.001 |
| **Temporal** | -3.5 (13.1) | 74.9 (44.0) | <0.001 |
| **Parietal** | -9.1 (14.4) | 78.8 (34.6) | <0.001 |
| **Striatum** | 31.7 (14.9) | 90.4 (33.4) | <0.001 |

Values are shown as median (IQR)

**Sup. Table 4. Regional neuropathological scores**

| **ROI** | **VR-** | **VR+** | ***p*** |
| --- | --- | --- | --- |
| **Anterior cingulte gyrus** | 0.0 (0.7) | 1.6 (0.6) | <0.001 |
| **Midfrontal lobe** | 0.1 (0.7) | 1.9 (1.2) | <0.001 |
| **Posterior cingulate gyrus** | 0.3 (0.8) | 1.6 (1.1) | <0.001 |
| **Precuneus** | 0.1 (0.7) | 1.9 (0.8) | <0.001 |
| **Superior temporal gyrus** | 0.4 (1.3) | 1.9 (1.5) | <0.01 |
| **Middle tempral gyrus** | 0.6 (1.4) | 2.3 (0.3) | <0.01 |
| **Inferior parietal lobe** | 0.5 (1.3) | 2.3 (0.5) | <0.001 |

Values are shown as median (IQR)

**Sup. Table 5. Overview of intra- and inter-reader agreement**Color scale in the second column refers to the Centiloid level, reflecting either a low amyloid burden (green), intermediate burden (yellow) or high burden (red)**.** Color scale in column 3-6 reflects final visual read classification as negative (green) or positive (red).

| **Subject** | **Centiloid** | **Reader 1**  **Initial read** | **Reader 1**  **Second read** | **Reader 2** | **Reader 3** | **Mean reader confidence** |
| --- | --- | --- | --- | --- | --- | --- |
| **1** | -15,95 | 0 | 0 | 0 | 0 | 4,25 |
| **2** | -10,15 | 0 | 0 | 0 | 0 | 4,25 |
| **3** | 0,78 | 0 | 0 | 0 | 0 | 4,5 |
| **4** | 1,48 | 0 | 0 | 0 | 0 | 3,75 |
| **5** | 1,53 | 0 | 0 | 0 | 0 | 4,5 |
| **6** | 1,60 | 0 | 0 | 0 | 0 | 4,75 |
| **7** | 2,47 | 1 | 0 | 0 | 0 | 3,5 |
| **8** | 3,20 | 1 | 0 | 0 | 0 | 5 |
| **9** | 5,02 | 1 | 0 | 0 | 0 | 3,75 |
| **10** | 6,12 | 0 | 0 | 0 | 0 | 3,75 |
| **11** | 6,31 | 0 | 0 | 0 | 0 | 4,5 |
| **12** | 6,89 | 1 | 0 | 0 | 1 | 3,75 |
| **13** | 7,20 | 0 | 0 | 0 | 0 | 4 |
| **14** | 7,73 | 0 | 0 | 0 | 0 | 4,75 |
| **15** | 7,97 | 0 | 0 | 0 | 0 | 3,75 |
| **16** | 8,14 | 1 | 0 | 0 | 0 | 3,75 |
| **17** | 8,44 | 0 | 0 | 0 | 0 | 3,5 |
| **18** | 8,48 | 0 | 0 | 0 | 0 | 4,75 |
| **19** | 8,60 | 0 | 0 | 0 | 0 | 5 |
| **20** | 9,81 | 0 | 0 | 0 | 0 | 4,5 |
| **21** | 10,08 | 0 | 0 | 0 | 0 | 3,75 |
| **22** | 10,32 | 0 | 0 | 0 | 0 | 4,5 |
| **23** | 10,69 | 0 | 0 | 0 | 0 | 4,75 |
| **24** | 10,92 | 0 | 0 | 0 | 0 | 4,5 |
| **25** | 10,93 | 1 | 0 | 1 | 0 | 3,75 |
| **26** | 11,16 | 1 | 1 | 1 | 1 | 4,5 |
| **27** | 11,34 | 1 | 1 | 0 | 0 | 4 |
| **28** | 11,58 | 1 | 0 | 0 | 1 | 3 |
| **29** | 11,71 | 0 | 0 | 0 | 0 | 4,5 |
| **30** | 12,01 | 1 | 0 | 0 | 0 | 3,25 |
| **31** | 12,09 | 0 | 0 | 0 | 0 | 4,5 |
| **32** | 12,10 | 1 | 1 | 0 | 0 | 2,75 |
| **33** | 12,12 | 0 | 0 | 0 | 0 | 4,5 |
| **34** | 12,37 | 0 | 1 | 0 | 0 | 2,75 |
| **35** | 12,80 | 0 | 0 | 0 | 0 | 3,5 |
| **36** | 13,39 | 1 | 1 | 1 | 1 | 4 |
| **37** | 13,51 | 0 | 0 | 0 | 0 | 3,75 |
| **38** | 13,57 | 0 | 0 | 0 | 0 | 4,5 |
| **39** | 13,93 | 1 | 1 | 1 | 1 | 4 |
| **40** | 13,96 | 0 | 0 | 0 | 0 | 4 |
| **41** | 14,38 | 1 | 0 | 0 | 0 | 4,25 |
| **42** | 15,57 | 0 | 0 | 0 | 0 | 4,75 |
| **43** | 16,24 | 0 | 0 | 0 | 0 | 4,5 |
| **44** | 16,67 | 0 | 0 | 0 | 1 | 4 |
| **45** | 16,97 | 1 | 1 | 1 | 0 | 3,5 |
| **46** | 16,97 | 0 | 0 | 0 | 0 | 4,75 |
| **47** | 17,05 | 0 | 0 | 1 | 0 | 4 |
| **48** | 17,21 | 1 | 1 | 1 | 0 | 3,25 |
| **49** | 17,35 | 0 | 0 | 0 | 0 | 3,75 |
| **50** | 18,63 | 1 | 1 | 1 | 0 | 3 |
| **51** | 19,00 | 0 | 0 | 0 | 0 | 4,5 |
| **52** | 19,00 | 1 | 1 | 1 | 1 | 3,5 |
| **53** | 19,25 | 0 | 0 | 0 | 0 | 3,5 |
| **54** | 19,97 | 0 | 0 | 0 | 0 | 5 |
| **55** | 20,22 | 0 | 0 | 0 | 0 | 3,5 |
| **56** | 20,38 | 1 | 1 | 1 | 1 | 4,5 |
| **57** | 20,40 | 1 | 1 | 1 | 1 | 3,5 |
| **58** | 20,76 | 1 | 1 | 1 | 1 | 4 |
| **59** | 21,17 | 1 | 1 | 1 | 1 | 3 |
| **60** | 22,05 | 1 | 1 | 0 | 1 | 4,5 |
| **61** | 23,02 | 1 | 1 | 1 | 1 | 3,25 |
| **62** | 23,29 | 1 | 1 | 1 | 1 | 4 |
| **63** | 23,83 | 0 | 1 | 0 | 1 | 3,5 |
| **64** | 25,15 | 1 | 1 | 1 | 1 | 4,25 |
| **65** | 25,25 | 1 | 1 | 0 | 1 | 4 |
| **66** | 25,34 | 1 | 1 | 0 | 0 | 3,25 |
| **67** | 25,77 | 1 | 1 | 1 | 1 | 3,5 |
| **68** | 26,12 | 1 | 1 | 1 | 1 | 4,75 |
| **69** | 27,44 | 1 | 1 | 1 | 1 | 4,25 |
| **70** | 27,62 | 1 | 1 | 1 | 0 | 3,75 |
| **71** | 27,88 | 1 | 1 | 1 | 1 | 3,75 |
| **72** | 29,20 | 1 | 1 | 1 | 1 | 4,25 |
| **73** | 32,29 | 1 | 1 | 1 | 1 | 4 |
| **74** | 32,94 | 1 | 1 | 1 | 1 | 4,25 |
| **75** | 32,95 | 1 | 1 | 1 | 1 | 4,5 |
| **76** | 35,20 | 1 | 1 | 1 | 1 | 4,75 |
| **77** | 35,59 | 1 | 1 | 1 | 1 | 4,5 |
| **78** | 35,63 | 1 | 1 | 1 | 1 | 4 |
| **79** | 36,93 | 1 | 1 | 1 | 1 | 4,25 |
| **80** | 38,45 | 1 | 1 | 1 | 1 | 4,75 |
| **81** | 39,28 | 1 | 1 | 1 | 1 | 5 |
| **82** | 41,41 | 1 | 1 | 1 | 1 | 4,5 |
| **83** | 46,97 | 1 | 1 | 1 | 1 | 4,5 |
| **84** | 47,17 | 1 | 1 | 1 | 1 | 4,75 |
| **85** | 47,17 | 1 | 1 | 1 | 1 | 4,25 |
| **86** | 48,47 | 1 | 1 | 1 | 1 | 4,5 |
| **87** | 52,26 | 1 | 1 | 1 | 1 | 5 |
| **88** | 53,38 | 1 | 1 | 1 | 1 | 5 |
| **89** | 55,77 | 1 | 1 | 1 | 1 | 4 |
| **90** | 57,43 | 1 | 1 | 1 | 1 | 4,75 |
| **91** | 59,75 | 1 | 1 | 1 | 1 | 4,25 |
| **92** | 62,10 | 1 | 1 | 1 | 1 | 5 |
| **93** | 63,20 | 1 | 1 | 1 | 1 | 5 |
| **94** | 64,11 | 1 | 1 | 1 | 1 | 4,75 |
| **95** | 64,11 | 1 | 1 | 1 | 1 | 4,25 |
| **96** | 64,51 | 1 | 1 | 1 | 1 | 4,5 |
| **97** | 69,12 | 1 | 1 | 1 | 1 | 5 |
| **98** | 69,43 | 1 | 1 | 1 | 1 | 5 |
| **99** | 75,81 | 1 | 1 | 1 | 1 | 5 |
| **100** | 79,34 | 1 | 1 | 1 | 1 | 5 |

|  | **Visual Read ROI** | | | | | | | | | | | | | | | |
| --- | --- | --- | --- | --- | --- | --- | --- | --- | --- | --- | --- | --- | --- | --- | --- | --- |
|  | **Global** | | **Frontal** | | | | **PC/PCC** | | | | **Parietal** | | **Temporal** | | | |
| **Neuropathology ROI** | **Mean of all ROIs** | | **MFL** | | **ACG** | | **PCG** | | **PRC** | | **IPL** | | **STG** | | **MTG** | |
|  | VR- | VR+ | VR- | VR+ | VR- | VR+ | VR- | VR+ | VR- | VR+ | VR- | VR+ | VR- | VR+ | VR- | VR+ |
| **mCERAD_SOT_ 0-1** | 12 (43%) | 1 (4%) | 13 (46%) | 2 (7%) | 12 (43%) | 2 (7%) | 12 (43%) | 2 (7%) | 12 (43%) | 1 (4%) | 12 (43%) | 0  (0%) | 14  (50%) | 1 (4%) | 15  (54%) | 0  (0%) |
| **mCERAD_SOT_ 1-1.5** | 1 (4%) | 5 (18%) | 1  (4%) | 3  (11%) | 2  (7%) | 3 (11%) | 2  (7%) | 4 (14%) | 2  (7%) | 4 (14%) | 5 (18%) | 2 (7%) | 5 (18%) | 0  (0%) | 2 (7%) | 1 (4%) |
| **mCERAD_SOT_ >1.5** | 0  (0%) | 9 (32%) | 1 (4%) | 8 (29%) | 1 (4%) | 8 (29%) | 1 (4%) | 7 (25%) | 1 (4%) | 8 (29%) | 3 (11%) | 6 (21%) | 3 (11%) | 5 (18%) | 5 (18%) | 5 (18%) |

**Sup. Table 6. Visual read against neuropathological scoring of neuritic plaque density**

PC/PCC: precuneus/posterior cingulate cortex; MFL: midfrontal lobe; ACG: anterior cingulate gyrus; PCG: posterior cingulate gyrus; PRC: precuneus; IPC: inferior parietal cortex; STG: superior temporal gyrus; MTG: middle temporal gyrus; LBD: lewy body dementia; AD: Alzheimer’s dementia

**Sup. Figure 1. Example images of harmonization process**

Image quality of the amyloid PET scans was different between the 2 cohorts in terms of resolution and noise level. Due to implementing TOF+PSF modeling in ALFA+ PET image reconstruction, these PET images were sharper compared to the ones in the ADC cohort. To perform the harmonization step, a subgroup of negative (example top row) and positive (example bottomrow) PET images in native space and normalized to the whole cerebellum were selected. The selected ALFA+ PET images were smoothed with different Gaussian kernel sizez, ranging from 3mm to 6 mm, and then visually compared with the negative/positive images from ADC cohort. An isotropic Gaussian filter with a FWHM of 4 mm produced the most comparable image quality in ALFA+ cohort. Thus, all PET images from this cohort were smoothed with this optimal filter prior visual assessment and for quantification.

**
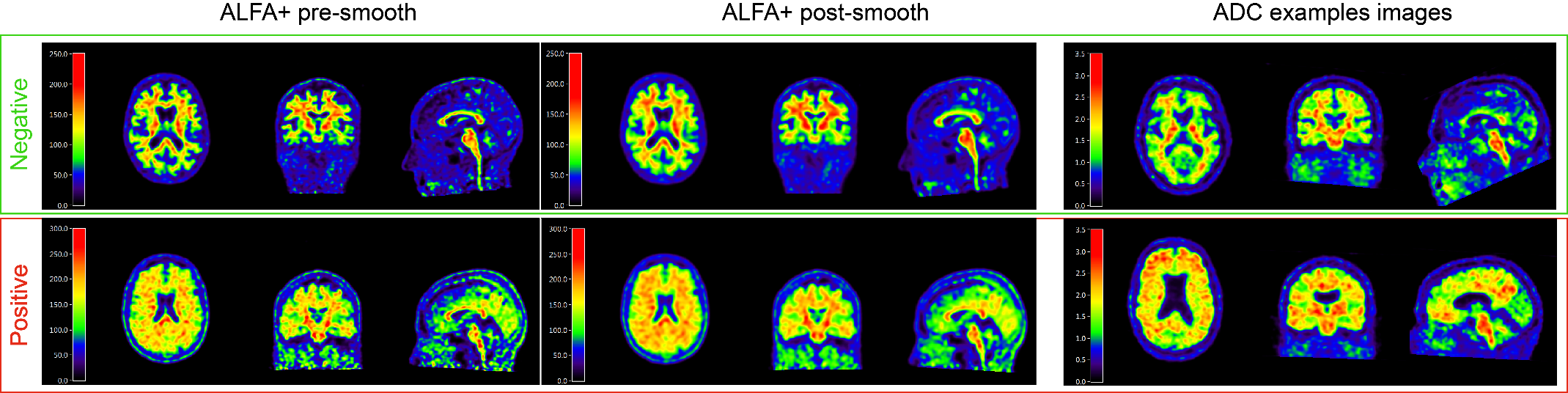
**

**Sup. Figure 2. Quantification ROIs based on visual read guidelines**

A total of 5 regions-of-interest were created using the Desikan-Killiany atlas to to reflect the visual assessment guidelines and enable regional quantification: 1) frontal (light blue): rostral and caudal anterior cingulate cortex, medial and lateral orbitofrontal, superior frontal, frontal pole, rostral and caudal middle frontal, pars orbitalis, pars triangularis, and pars opercularis; 2) the precuneus (PC)/posterior cingulate cortex (PCC) (dark blue): precuneus, posterior cingulate cortex, and isthmus cingulate cortex; 3) lateral-parietal (red): superior parietal, supramarginal, and inferior parietal; 4) lateral temporal (orange): transverse temporal, temporal pole and inferior, middle, and superior temporal cortex; and finally 5) striatum (yellow): putamen and caudate nucleus.

**
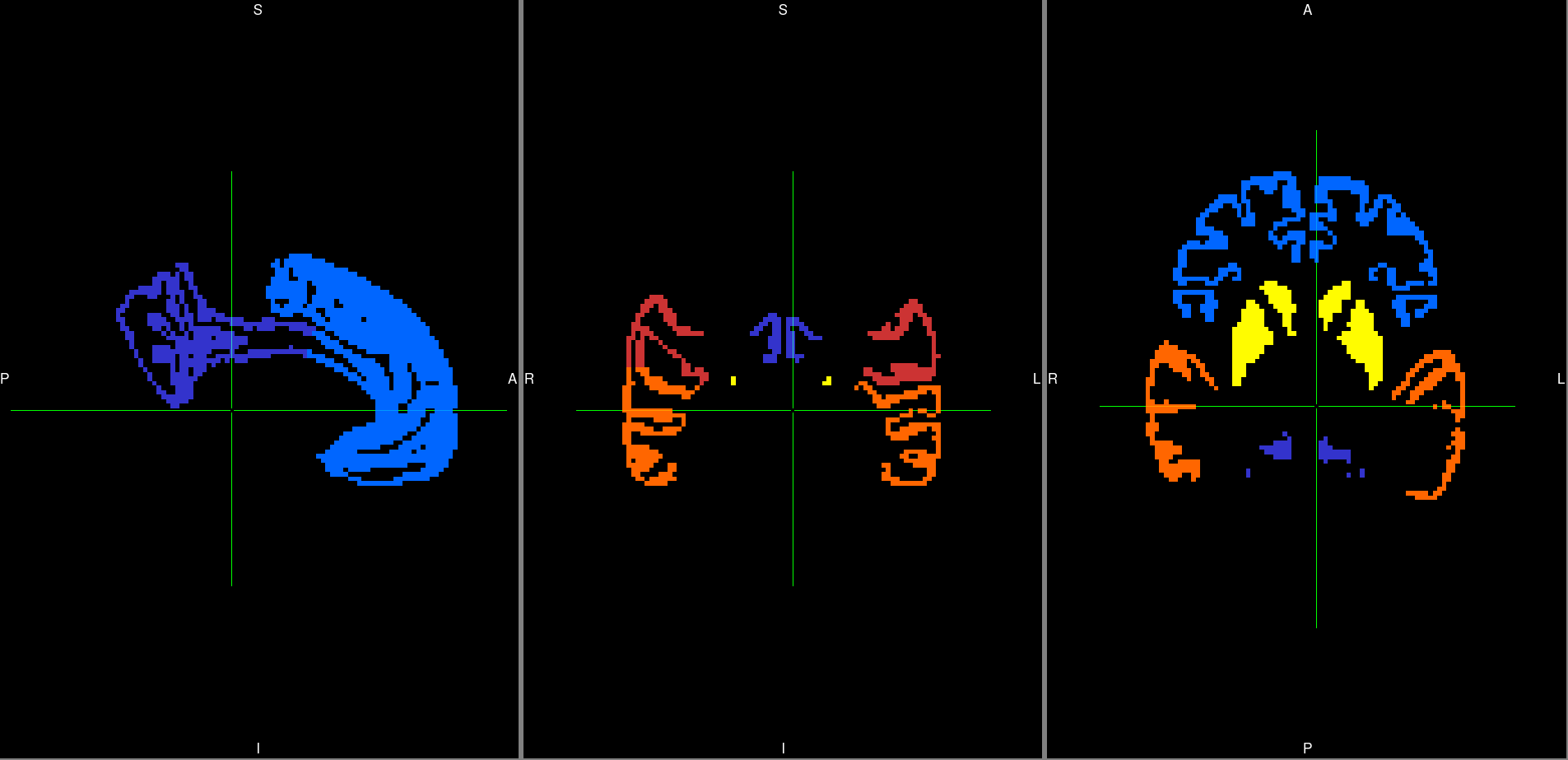
**


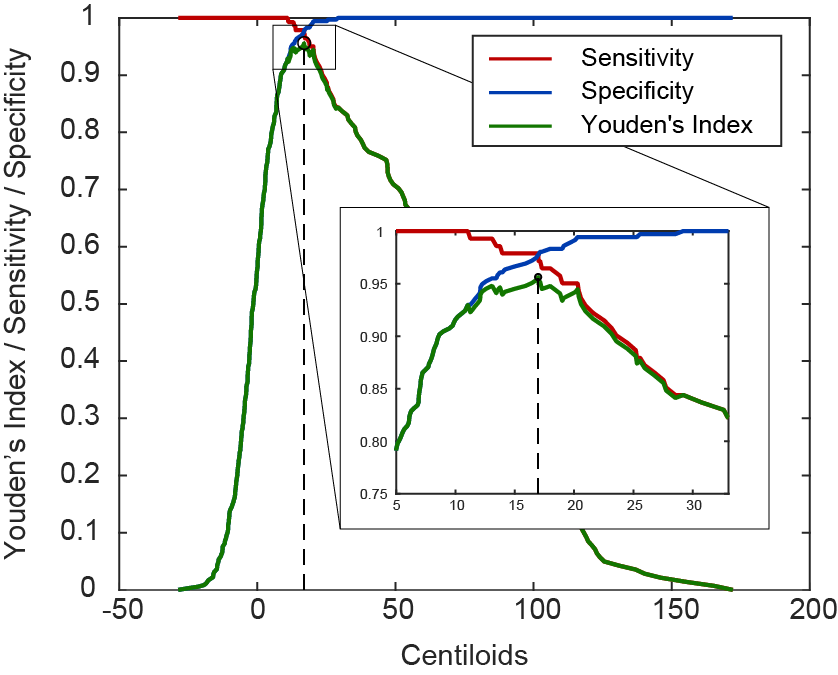


**Sup. Figure 3. Results of ROC analyses with VR as the reference.**

Sensitivity, specificity, and Youden Index as a function of CL are shown. The dotted line represents the optimal Youden Index corresponding to a CL=17 cut-off.

**Visual read stages against Centiloid and clinical diagnosis**

VR stages were associated with CL groups of low, gray-zone, and high amyloid burden. More specifically, VR- subjects nearly all (93.8%) had a low CL burden and only 22 (6.2%) of these subjects had a CL value within the gray-zone, but not above 30. A CL burden >12 was observed in 95.5%, 100%, and 100% for VR stage 1, 2 and 3, respectively (**Sup. Figure 4**).

Scans classified as VR- were most often (86.5%) from CU subjects, and the relative contribution of CU subjects was lowest for VR stage 3 (19.1%). In turn, 82 (74.5%) of VR+ stage 3 subjects had a clinical diagnosis of AD dementia. Most non-AD dementia subjects had no amyloid burden (40/48, 83.3%), with a subset showing early accumulation (VR+ stage 1: 2/48, 4.2%), or more advanced amyloid burden (VR+ stage 3: 6/48, 12.5%). More specifically, VR- negative non-AD dementia subjects mainly consisted of FTD cases (*N*=20, 50%) and most non-AD dementia stage 2 or stage 3 cases were clinically diagnosed as Dementie with Lewy Bodies (DLB) (*N*=4, 50%). Finally, the VR+ stage 1 group did not include clinical demented cases (**Sup. Figure 4**). Note, clinical diagnosis was made pre-PET disclosure.

**Sup. Figure 4**

**
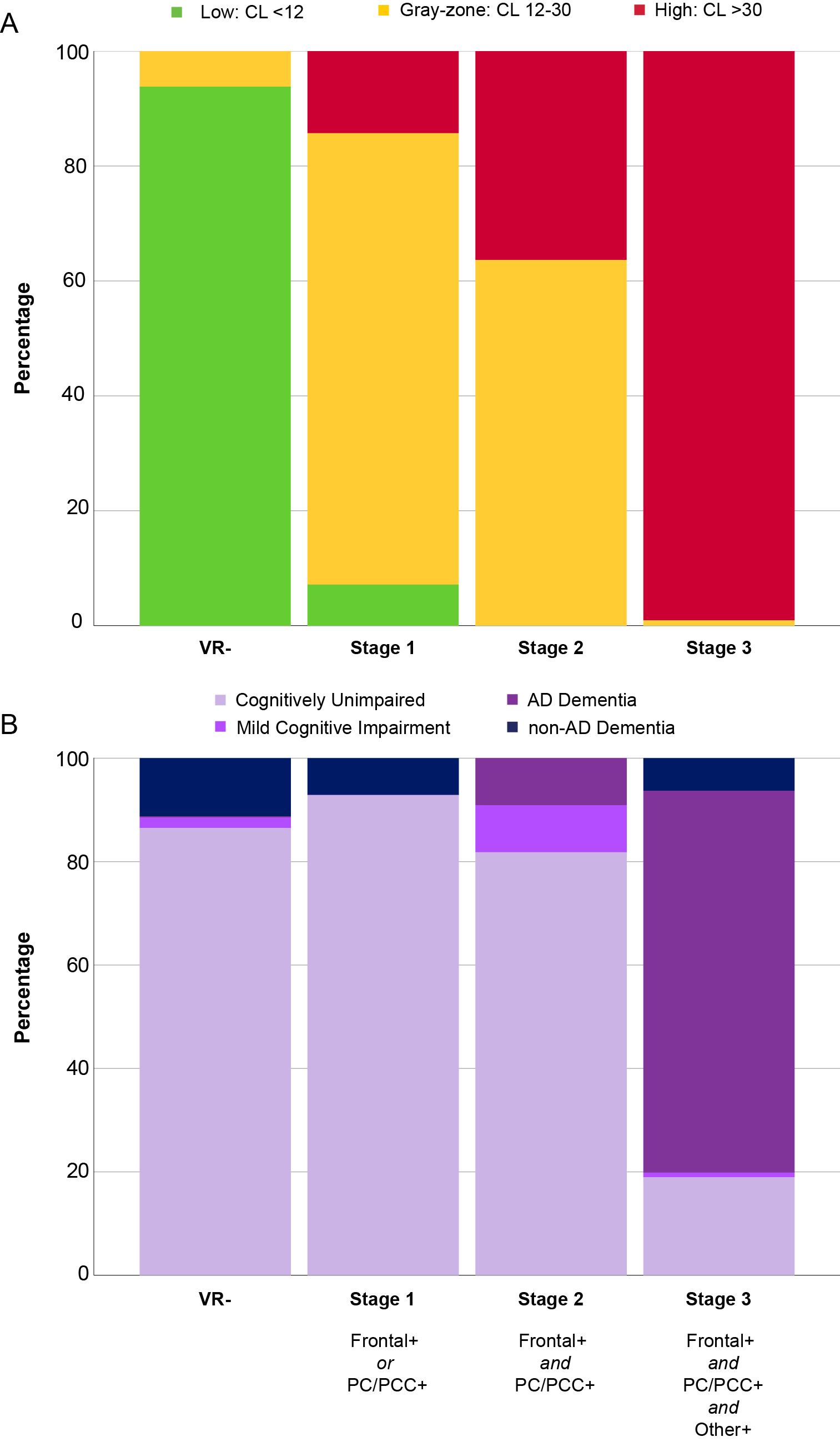
**

**Sup. Figure 4. Percantage of amyloid status and diagnosis per VR staging.**

Bar graphs represent the percentage distribution A) of CL groups and B) clinical diagnosis across visual read stages. Clinical Diagnoses were done before PET disclosure.

**References**

1. Ten Kate M, Dicks E, Visser PJ, van der Flier WM, Teunissen CE, Barkhof F, et al. Atrophy subtypes in prodromal Alzheimer's disease are associated with cognitive decline. Brain. 2018;141:3443-56. doi:10.1093/brain/awy264.

2. Ten Kate M, Barkhof F, Visser PJ, Teunissen CE, Scheltens P, van der Flier WM, et al. Amyloid-independent atrophy patterns predict time to progression to dementia in mild cognitive impairment. Alzheimers Res Ther. 2017;9:73. doi:10.1186/s13195-017-0299-x.
